# Comparison of fruit organic acids and metabolism-related gene expression between *Cerasus humilis* (Bge.) Sok and *Cerasus glandulosa* (Thunb.) Lois

**DOI:** 10.1371/journal.pone.0196537

**Published:** 2018-04-26

**Authors:** Xiaopeng Mu, Pengfei Wang, Junjie Du, Yu Gary Gao, Jiancheng Zhang

**Affiliations:** 1 College of Horticulture, Shanxi Agricultural University, Taigu, Shanxi, P. R. China; 2 Shanxi Key Laboratory of Germplasm Improvement and Utilization in Pomology, Taigu, Shanxi, P. R. China; 3 OSU South Centers, College of Food, Agricultural, and Environmental Sciences, The Ohio State University, Piketon, Ohio, United States of America; Key Laboratory of Horticultural Plant Biology (MOE), CHINA

## Abstract

*Cerasus humilis* (Bge.) Sok and *Cerasus glandulosa* (Thunb.) Lois are economically important fruit-producing shrubs. Although these two species have similar looking fruits, their fruit organic acid contents differ drastically. In this study, we focused on comparing the organic acid content, activity of enzymes gene expression involved in organic acid metabolism in both *C*. *humilis* and *C*. *glandulosa* fruits. To investigate the differences of organic acid metabolism in fruits of these two species, a comparative transcriptome analysis was performed. Our results showed that temporal changes of two main organic acids exhibited different trends in these two species. Transcriptome sequencing of developing *C*. *humilis* and *C*. *glandulosa* fruits systematically revealed 6,594 differentially expressed genes. Compared with *C*. *humilis*, the expression levels of 3,469 and 3,125 genes were up- and down-regulated in *C*. *glandulosa*, respectively, including one PEPC gene, 12 malic acid metabolism genes, 25 citric acid cycle genes, and 194 NAD/NADP metabolism genes. The correlation analysis and principal component analysis of gene expression, enzymatic activity and organic acid content showed that differences in the expression of genes encoding the NAD-malate dehydrogenase (*NAD-MDH*) and NADP-malate enzyme (*NADP-ME*) contributed substantially to the observed differences in organic acid accumulation of two species. Our results provide a solid foundation for future elucidation of key mechanisms regulating organic acid biosynthesis in *C*. *humilis* and *C*. *glandulosa* fruits and could lead to efficient and highly targeted generation of more commercially accepted cultivars.

## Introduction

The acidity of fleshy fruit is an important component of fruit organoleptic quality [1,2]. Fruit acidity is due to the presence of organic acids synthesized within fruits [3], with considerable changes in production during different developmental stages [4–6]. The variations in the production of organic acids during different developmental stages vary drastically among species. For example, the main organic acids in ‘Honeycrisp’ apple flesh increase gradually with fruit development [6], while the content of the main organic acids in sand pear fruits decline with fruit development [7].

Organic acids could be utilized as the substrates in the glycolytic pathway, trimesic acid cycle, and gluconeogenesis during fruit maturation process [8]. Therefore, fruit organic acid content is closely associated with changes in the activities of related enzymes. For example, phosphoenolpyruvate carboxylase (PEPC), NAD-dependent malate dehydrogenase (NAD-MDH), and NADP-malic enzyme (NADP-ME) are important for the synthesis of malic acid and citric acid [9–12]. Previous studies revealed that PEPC activity is positively correlated with navel orange organic acid content [13], while the *PEPC* gene expression level and associated protein content help regulate peach and pineapple fruit acidity [14]. Additionally, PEPC activity is also affected by malic acid, pyruvic acid, cytoplasmic pH, and reversible phosphorylation during fruit organic acid metabolism [15–17]. Thus, the effects of PEPC on fruit organic acid metabolism are complex. Furthermore, NAD-MDH activity is positively correlated with malic acid content in nectarines [18], while the expression of the *NAD-MDH* gene in peach and apple does not directly affect fruit acidity [19,20]. In most fruits, NADP-ME plays a major role in the degradation of malic acid. The organic acid content of loquat fruit is negatively correlated with NADP-ME activity [18]. In grapes, the expression level of the *NADP-ME* gene is relatively high in the young fruits, declines in the maturing fruits, and increases when the fruits mature [21].

The genetic regulation of organic acids is complex and most often involves polygenes. However, the organic acid content of some plant species that produce moderately or highly acidic fruits is controlled by one major gene (D model), with low acidity being a recessive trait [8]. Previous studies concluded there is no correlation or no significant correlation between the expression of organic acid metabolism-related genes and organic acid content [14,19,22]. However, it was reported that the expression of the cytosolic *NADP-IDH* gene in lemon fruits is well correlated with changes in NADP-IDH (NADP^+^ -isocitrate dehydrogenase) activity and promotes the degradation of organic acids [23].

*Cerasus humilis* is a small shrub grown in China for its fruit [24]. It is highly stress-resistant, especially to drought and low temperatures [25,26]. Additionally, its fruit contains more calcium, flavonoids, vitamins, and other nutrients than many other fruits [27], making it a very promising multi-purpose fruit tree species. It is rare for *C*. *humilis* cultivars to produce fruits with low acidity, which greatly limits the commercial potential of this species [28]. *Cerasus glandulosa* is closely related to *C*. *humilis* but produces fruits with lower organic acid content [29]. There have been no studies that examined the effects of enzyme activities and gene expression levels on organic acid content in *C*. *humilis* or *C*. *glandulosa* fruits. In this study, we investigated the temporal changes in organic acid content, the activities of key enzymes, and the expression levels of genes associated with malic acid metabolism in the developing fruits of *C*. *humilis* and *C*. *glandulosa*. Our results should be useful for improving the fruit quality of *C*. *humilis* and *C*. *glandulosa*, and for characterizing the molecular mechanisms regulating fruit organic acid biosynthesis.

## Materials and methods

The fruits of *C*. *humilis* (cv. Jinou 1; high acidity) and *C*. *glandulosa* (cv. DS-1; low acidity) were used as the test materials in this study. Because of the asynchrony in the developmental processes of *C*. *humilis* and *C*. *glandulosa* fruits, *C*. *humilis* fruit samples were collected at seven developmental stages (8, 10, 12, 14, 16, 18, and 19 weeks after flowering), while *C*. *glandulosa* fruit samples were collected at five developmental stages (8, 10, 12, 13, and 14 weeks after flowering), from plants grown at the experimental farm of the Horticultural College, Shanxi Agricultural University, Taigu, China. Following collection, the fruit samples were immediately frozen in liquid nitrogen and stored at -80 °C until analysis.

### Determination of total acid, malic acid, and citric acid contents

Fruit organic acid extraction and determination were carried out with some modifications to the methods described by Nergiz and Ergönül [30]. Samples were analyzed with HPLC (UltiMate3000 system, Thermo Fisher, USA), and the analysis conditions were as follows: chromatographic separation was performed on a Syncronis C18 column (5 μm, 250 mm × 4.6 mm); mobile phase was methanol:0.01mol/L K_2_HPO_4_ solution (10:90, pH = 2.0) with a flow rate of 0.5 mL/min; column temperature was set at 25 °C; sample injection volume was 15 μL; detection wavelength was set at 210 nm. Three replicates of each sample were analyzed.

### Determination of enzyme activity

Enzyme samples were prepared as described by Chen et al [8]. Enzyme activity was determined as described by Hirai and Ueno [31], but the reaction volume was changed to 3 mL. The enzyme activity of NAD-MDH was measured in reaction mixture containing 300 μL Tris-HCl (800 mM/L, pH 8.2), 150 μL KHCO_3_ (800 mM/L), 150 μL MgCl_2_ (40 mM/L), 150 μL GSH (10 mM/L), 150 μL NADH (3 mM/L), 1600 μL OAA (4 mM/L), and 500 μL enzyme extract; The enzyme activity of NADP-ME was measured in reaction mixture containing 300 μL Tris-HCl (800 mM/L, pH 7.4), 150 μL MnSO_4_ (4 mM/L), 150 μL NADP (3.4 mM/L), 300 μL ddH_2_O, 1600 μL malic acid (4 mM/L), and 500 μL enzyme extract; The enzyme activity of PEPC was measured in reaction mixture containing 300 μL Tris-HCl (800 mM/L, pH 8.5), 150 μL KHCO_3_ (200 mM/L), 150 μL MgCl_2_ (40 mM/L), 150 μL GSH (10 mM/L),150 μL NADH (3 mM/L), 1600 μL PEP (4 mM/L), and 500 μL enzyme extract. The reaction components were mixed just before adding the substrate. The absorbance (at 340 nm) of the enzyme samples was measured using a UV-2450 spectrophotometer (Shimadzu Corporation, Japan) immediately after adding the substrate (1 s for the unit readings with a 3-min scan). Changes in absorbance were recorded and the analysis was repeated three times. One enzyme unit corresponded to an absorbance change of 0.01 in 1 min, and enzyme activity was expressed in units per gram of pulp (fresh weight) per minute (U g^−1^ FW min^−1^).

### RNA extraction

Frozen fruit samples (1 g) from each of the different developmental stages (seven stages for *C*.*humilis* and five stages for *C*.*glandulosa*) were used for the total RNA extraction using a modified CTAB method [27]. RNA quality was then assessed on 1% agarose gels for degradation and contamination. The purity of the extracted RNA was checked using the NanoPhotometer^®^ spectrophotometer (Implen, CA, USA), while the concentration was measured using the Qubit^®^ RNA Assay Kit and the Qubit^®^ 2.0 Fluorometer (Life Technologies, CA, USA). Finally, RNA integrity was assessed using the RNA Nano 6000 Assay Kit and the 2100 Bioanalyzer system (Agilent Technologies, CA, USA).

### Library preparation for transcriptome sequencing

The sequencing cDNA libraries were constructed for *C*.*humilis* and *C*.*glandulosa* respectively with the NEBNext^®^ Ultra^™^ RNA Library Prep Kit for Illumina^®^ (NEB, USA) following the manufacturer’s recommendations. Index codes were added to attribute sequences to each sample. Briefly, mRNA was purified from total RNA using poly-T oligo-attached magnetic beads and then fragmented by heating in 5X NEBNext First Strand Synthesis Reaction Buffer containing divalent cations. First-strand cDNA was synthesized using a random hexamer primer and M-MuLV Reverse Transcriptase (RNase H−). Second-strand cDNA was synthesized with DNA Polymerase I and RNase H. The remaining overhanging ends were converted to blunt ends via exonuclease/polymerase activities. After adenylating the 3′ ends of cDNA fragments, the NEBNext Adaptor with a hairpin loop structure was ligated for a subsequent hybridization. To select cDNA fragments that were 150–200 bp long, the library fragments were purified with the AMPure XP system (Beckman Coulter, Beverly, USA). Then, 3 μL USER enzyme (NEB, USA) was added to the size-selected, adaptor-ligated cDNA samples, which were then incubated at 37 °C for 15 min and then at 95 °C for 5 min. The subsequent polymerase chain reaction (PCR) was completed with the Phusion High-Fidelity DNA polymerase, universal PCR primers, and the Index (X) Primer. Finally, the PCR products were purified with the AMPure XP system and the library quality was assessed using the 2100 Bioanalyzer system.

### Clustering and sequencing

The index-coded samples were clustered using the cBot Cluster Generation System and the TruSeq PE Cluster Kit v4 cBot-HS (Illumina) according to the manufacturer’s instructions. The libraries were then sequenced with the HiSeq 2500 platform (Illumina) to generate paired-end reads.

Raw data (raw reads) in fastq format were first processed using in-house Perl scripts. Clean data (clean reads) were obtained by removing reads with an adaptor, reads containing multiple Ns, and low-quality reads. Additionally, the Q20, Q30, GC content, and sequence duplication level of the clean data were calculated. All downstream analyses were based on high quality clean data.

### Transcriptome assembly and function annotation

For all libraries/samples, the left files (read1 files) were pooled into one left.fq file, while the right files (read2 files) were combined into one right.fq file. The transcriptome was assembled based on the left.fq and right.fq files using Trinity [32] with min_kmer_cov set to 2 by default. All other parameters were also set to default values. The transcriptome data of *C*. *humilis* and *C*. *glandulosa* were submitted to NCBI BioProject under the accession number PRJNA417674.

Gene function was annotated based on the following databases: Nr (NCBI non-redundant protein sequences); Nt (NCBI non-redundant nucleotide sequences); Pfam (Protein family); KOG/COG (Clusters of Orthologous Groups of proteins); Swiss-Prot (manually annotated and reviewed protein sequence database); KO (KEGG Ortholog database); and GO (Gene Ontology).

### Quantification of gene expression levels and identification of differentially expressed genes

Gene expression levels were estimated by RSEM [33] using a two-step process. First, clean data were mapped back to the assembled transcriptome. Second, the read count for each gene was obtained based on the mapping results.

For samples with three biological replicates, the analysis of differentially expressed genes between two conditions/groups was completed using the DESeq R package (1.10.1), which detects differentially expressed genes using a model based on the negative binomial distribution. The resulting P-values were adjusted with the Benjamini and Yekutieli approach for controlling the false discovery rate [34]. Genes with an adjusted P-value < 0.05 were considered differentially expressed. Prior to the analysis of differentially expressed genes, the read counts for each sequenced library were adjusted by the edgeR program package with one scaling normalization factor. Genes differentially expressed between two samples were analyzed with the DEGseq (2010) R package. The P-value was adjusted using the q-value [35]. The following threshold was applied to identify differentially expressed genes: q-value < 0.005 and |log_2_ (fold change)| ≥ 1. The WEGO online program (http://wego.genomics.org.cn/cgi-bin/wego/index.pl) was used for the GO functional classification of all unigenes and determination of the distribution of gene functions.

### Quantitative real-time PCR analysis

Total RNA was used as the template to synthesize cDNA with the PrimeScript^™^ RT reagent Kit with gDNA Eraser (Perfect Real Time) (TaKaRa, Japan). The 20-μL reverse transcription reaction mixture contained 5 μL total RNA (1 μg), 1 μL 10 mM dNTP, 1 μL random primer (9-mer) (50 mM), 1 μL oligo-d(T)_18_ primer (50 mM) (TaKaRa, Japan), and 6 μL DEPC water. The quantitative real-time PCR (qPCR) was completed using qPCR SYBR^®^ Premix Ex Taq^™^ II (Tli RNaseH Plus) (TaKaRa) and the 7500 Real-Time PCR System (Applied Biosystems, USA). The qPCR primers used to validate the differentially expressed genes were synthesized by Beijing Liuhe Genomics Technology Co., Ltd. (Table 1). Each gene was analyzed using four replicates, after which the average threshold cycle was calculated for each sample. An endogenous actin gene was used for data normalization [36]. Relative fold changes in gene expression were calculated using the 2^−ΔΔCt^ method.

**Table 1:** Primers used for quantitative real-time PCR.

Primer	Forward 5′-3′	Reverse 5′-3′	Product lengths (bp)
qChNAD-MDH	ACTGTTCAACAACGTGGTGC	TTCCAAGTACCCAATCACGA	106
qChNADP-ME	TATTGGATCATCCGGTGTTG	CAGACTGAGATGTCGGGTTG	113
qChPEPC	TCCTCCTCCGCGACTCCTA	CGCTGGCTTGCTTGTTGTT	140
qChCS	TGCTCACAGTGGAGTTCTGT	GCCTTTCAAGAGGCAAACCA	140
qCgNAD-MDH	CGATGCTGCTTTCCCTCTT	GAAGCCACCAACCATAACG	97
qCgNADP-ME	TATTGGATCATCCGGTGTTG	CAGACTGAGATGTCGGGTTG	113
qCgPEPC	TCCTCCTCCGCGACTCCTA	CGCTGGCTTGCTTGTTGTT	140
qCgCS	TGGGAAGGTTGTTCCTGGTT	AGCTTGGAGACCAGCTGAAA	128
qActin	ATCTGCTGGAAGGTGCTGAG	CCAAGCAGCATGAAGATCAA	100

Ch: *C*.*humilis*; cg: *C*.*glandulosa*; NAD-MDH: NAD-dependent Malate dehydrogenase; NADP-ME: NADP-dependent Malic enzyme; PEPC: Phosphoenolpyruvate carboxylase; CS: Citrate synthase.

### Statistical analysis

Results are expressed here as mean ± SD. The differences among means were evaluated using Tukey’s multiple comparison test and the statistical significance was set at *p* < 0.05. Principal component analysis (PCA) was carried out using SAS 8.0 (SAS Institute, Cary, NC, USA).

## Results

### Analysis of organic acid content in developing *C*. *humilis* and *C*. *glandulosa* fruits

**Fig 1:**
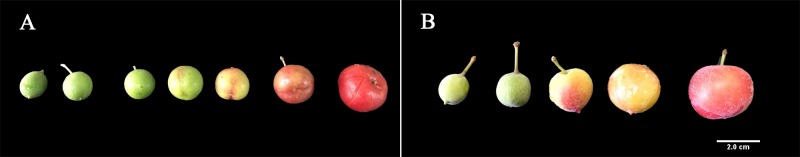
*C*. *humilis* (A: 8, 10, 12, 14, 16, 18, and 19 weeks after flowering) and *C*. *glandulosa* (B: 8, 10, 12, 13, and 14 weeks after flowering) fruits at different developmental stages.

The *C*. *humilis* and *C*. *glandulosa* fruits reached maturity at 19 and 14 weeks after flowering (WAF), respectively (Fig 1). Malic acid and citric acid were the main organic acids in their fruits. Similar trends were observed for the total acid, malic acid, and citric acid content of *C*. *humilis* fruits, with an increase during the early fruit-developmental stage, followed by a rapid increase during the fruit-enlargement stage (FES), and then a decrease in the mature fruits. The highest total acid (28.62±1.05 mg/g), malic acid (23.81±1.20 mg/g), and citric acid (3.53±0.23 mg/g) content were observed at 18 WAF. In mature fruits, the total acid, malic acid, and citric acid content decreased to 22.58±1.07 mg/g, 19.27±0.98 mg/g, and 2.48±0.21 mg/g, respectively (Table 2). Moreover, the malic acid and citric acid content of mature fruits accounted for 85.34% and 10.98% of the total acid content, respectively.

**Table 2:** Changes to total acid, malic acid, and citric acid contents in *C*. *humilis* and *C*. *glandulosa* fruits during development.

WAF	*C*. *humilis*			*C*. *glandulosa*		
	Total acid (mg/g FW)	Malic (mg/g FW)	Citric (mg/g FW)	Total acid (mg/g FW)	Malic (mg/g FW)	Citric (mg/g FW)
8	8.11±0.24	4.38±0.21	0.28±0.02	17.59±0.88	14.13±0.33	3.15±0.18
10	11.37±0.25	10.19±0.28	0.42±0.03	14.51±0.40	11.63±0.20	2.77±0.25
12	13.41±0.34	11.51±0.33	0.52±0.03	10.22±0.32	8.56±0.29	1.07±0.11
13	-	-	-	7.65±0.21	6.12±0.23	0.9±0.08
14	17.92±0.86	16.13±0.68	1.73±0.12	7.39±0.28	6.57±0.22	0.35±0.04
16	19.34±0.93	16.7±0.75	2.04±0.18	-	-	-
18	28.62±1.05	23.81±1.20	3.53±0.23	-	-	-
19	22.58±1.07	19.27±0.98	2.48±0.21	-	-	-

“-” means data is not available; WAF, weeks after flowering.

Similar trends among the total acid, malic acid, and citric acid content were also observed for *C*. *glandulosa* fruits. The total acid and citric acid content decreased throughout the fruit development period, while the malic acid content increased slightly before fruit maturation. Total acid (17.59±0.88 mg/g), malic acid (14.13±0.33 mg/g), and citric acid (3.15±0.18 mg/g) content were highest at 8 WAF. The total acid, malic acid, and citric acid content decreased to 7.39 mg/g, 6.57 mg/g, and 0.35 mg/g in mature fruits, respectively. The lowest malic acid content (6.12 mg/g) was recorded at 13 WAF (Table 2). In mature *C*. *glandulosa* fruits, malic acid and citric acid accounted for 88.90% and 4.74% of the total acid content, respectively.

### Analysis of organic acid-related enzyme activities in developing *C*. *humilis* and *C*. *glandulosa* fruits

**Fig 2:**
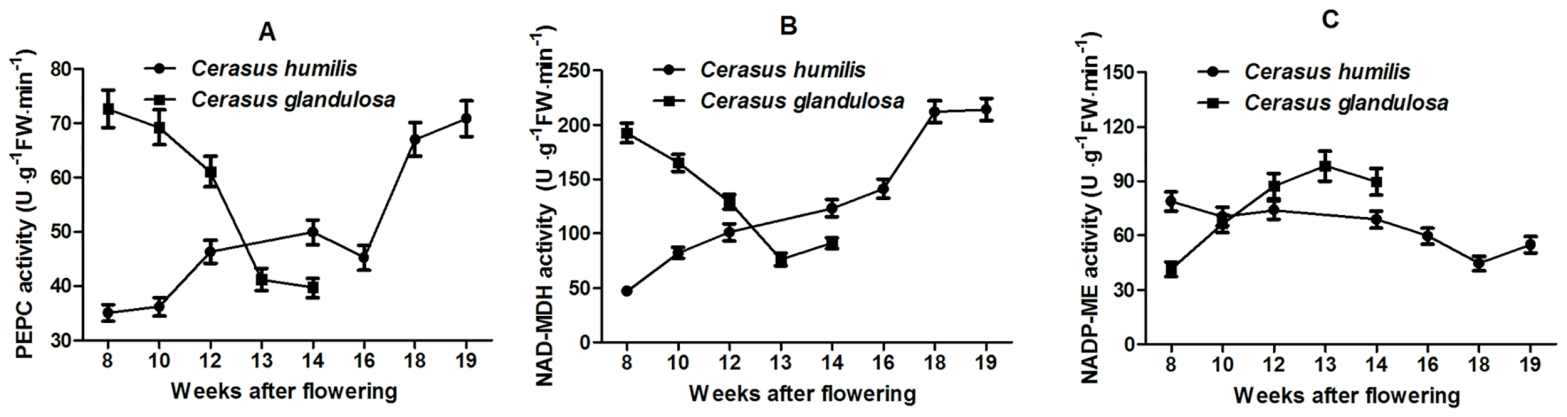
Changes in PEPC (A), NAD-MDH (B), and NADP-ME (C) activities in *C*. *humilis* and *C*. *glandulosa* during fruit development.

Phosphoenolpyruvate carboxylase (PEPC) catalyzes the β-carboxylation of PEP to produce oxaloacetic acid (OAA) and inorganic phosphoric acid. The OAA is reduced to malic acid in a reaction catalyzed by NAD-MDH [11]. The activity of PEPC in *C*. *humilis* fruits increased gradually from 8 to 14 WAF, then decreased slightly from 14 to 16 WAF, followed by a rapid increase to the maximum value of 70.91 U g^−1^ FW min^−1^ (Fig 2A). In contrast, in *C*. *glandulosa* fruits, the PEPC activity was 72.69 U g^−1^ FW min^−1^ at 8 WAF and then decreased to 39.75 U g^−1^ FW min^−1^ at maturity (Fig 2A).

NAD-MDH activity exhibited the opposite trend in the developing fruits of *C*. *humilis* and *C*. *glandulosa* (Fig 2B). In *C*. *humilis*, NAD-MDH activity at 8 WAF was 82.43 U g^−1^ FW min^−^1, which then gradually increased to 214.32 U g^−1^ FW min^−1^ as the fruits developed. In *C*. *glandulosa*, NAD-MDH activity was 192.69 U g^−1^ FW min^−1^ at 8 WAF, which then gradually decreased to the minimum value of 76.46 U g^−1^ FW min^−1^ at 13 WAF, after which the activity slowly increased to 91.25 U g^−1^ FW min^−1^ in mature fruits.

In developing *C*. *humilis* fruits, the activity of NADP-ME exhibited a decreasing trend from 8 to 18 WAF and then increased as the fruits matured (Fig 2C). Meanwhile, NADP-ME activity in *C*. *glandulosa* fruits gradually increased from 8 to 13 WAF, followed by a slight decrease as the fruits matured.

### Transcriptome sequencing and identification of differentially expressed genes

We obtained 38,969 unigenes following the transcriptome sequencing of *C*. *glandulosa* and *C*. *humilis* (S1 Table), including 6,594 differentially expressed genes. There were 3,469 up-regulated and 3,125 down-regulated genes in *C*. *glandulosa* compared with that in *C*. *humilis*, including 12 genes directly involved in malic acid metabolism (CL4433.Contig2_All, Unigene8844_All, CL188.Contig2_All, Unigene6244_All, Unigene18125_All, CL471.Contig2_All, CL738.Contig1_All, CL3719.Contig1_All, CL4438.Contig1_All, Unigene5127_All, CL4732.Contig2_All, and Unigene12965_All), 25 genes directly involved in the citric acid cycle, 194 genes involved in NAD/NADP metabolism, and one PEPC gene (CL2688.Contig3_All) (S2 Table).

**Fig 3:**
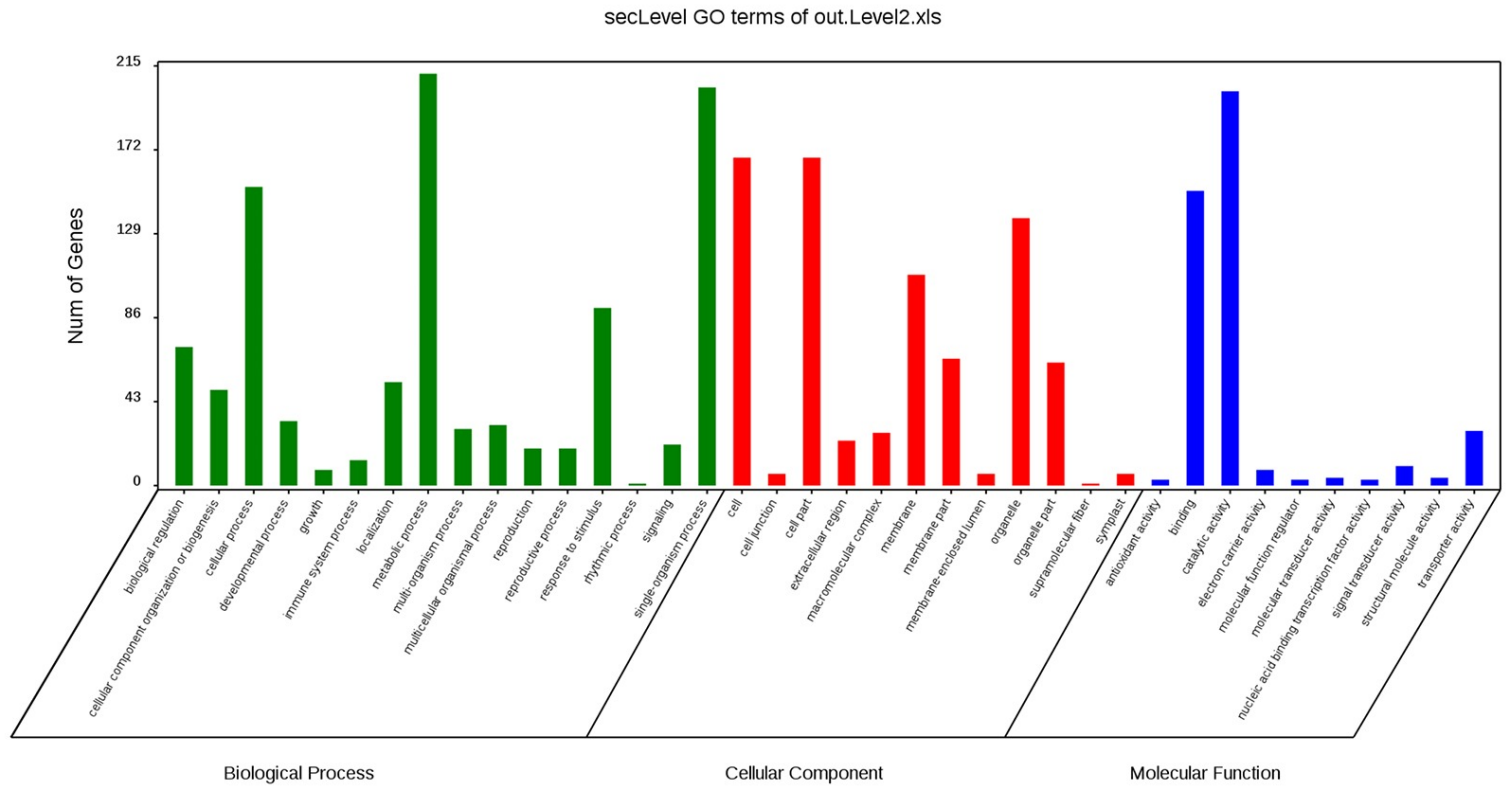
Gene Ontology classifications of the differentially expressed genes related to malic acid and citric acid metabolism.

The above-mentioned 223 genes involved in malic acid metabolism and citric acid metabolism were classified into GO categories (Fig 3). There were 16, 10, and 12 subclasses in the biological process, molecular function, and cellular component categories, respectively. In the biological process category, the metabolic process (211 genes), single-organism process (204 genes), and cellular process (153 genes) subgroups contained the most genes, while the rhythmic process (one gene) contained the fewest genes. In the molecular function category, the catalytic activity (202 genes) and binding (151 genes) subgroups contained the most genes, while the nucleic acid binding transcription factor activity, antioxidant activity, and molecular function regulator subgroups contained the fewest genes, each with only three genes. In the cellular component category, the cell part and cell subgroups included the most genes, both with 168 genes, while the supramolecular fiber subgroup contained only one gene.

**Fig 4:**
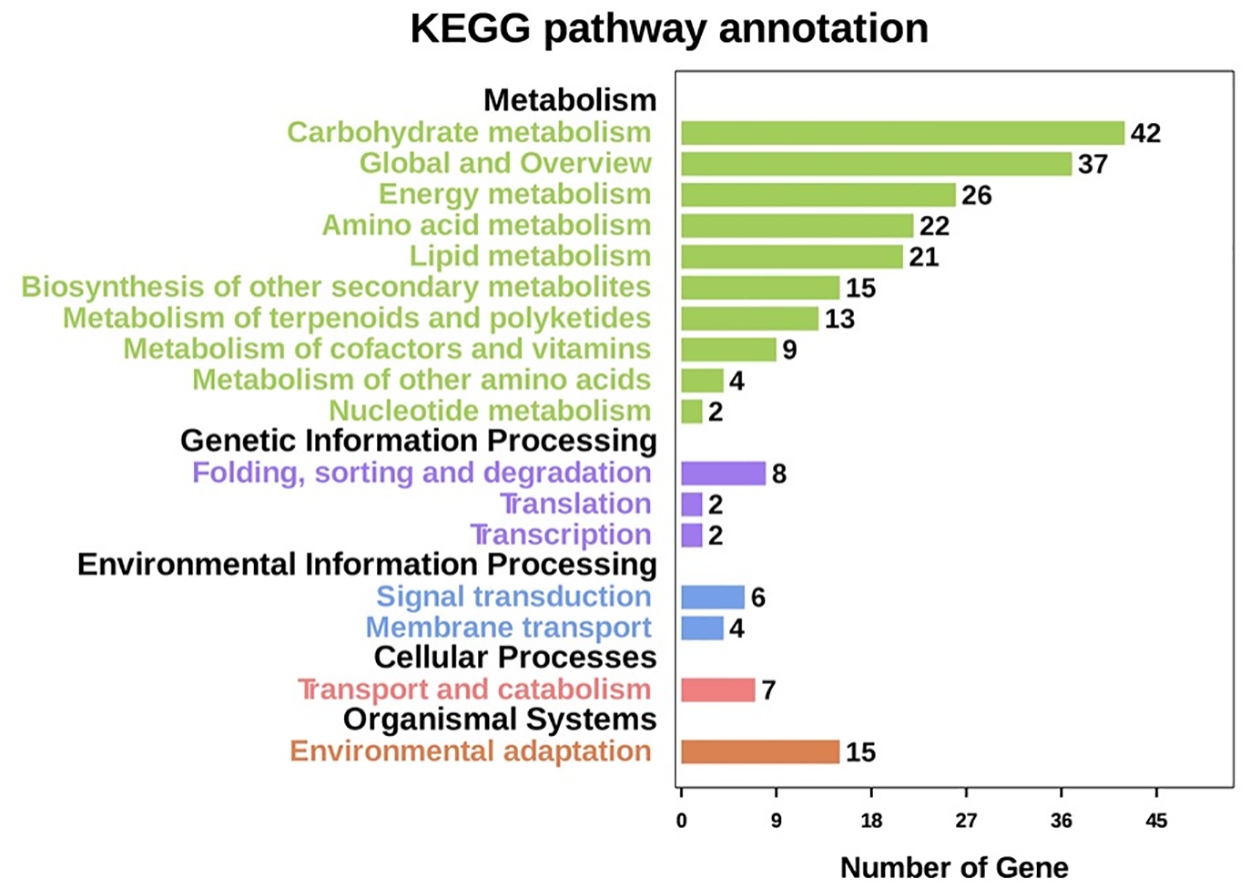
KEGG pathway classifications of the differentially expressed genes related to malic acid and citric acid metabolism.

The KEGG pathway analysis indicated that 233 differentially expressed genes were involved in 10 metabolic pathways, with the carbohydrate metabolism (42 genes), global and overview (37 genes), and energy metabolism (26 genes) pathways associated with the most genes. The pathways with the fewest genes were the metabolism of other amino acids (four genes) and nucleotide metabolism (two genes). The genetic information processing group contained folding, sorting, and degradation, as well as translation and transcription metabolic pathways. The environmental information processing group comprised signal transduction and membrane transport metabolic pathways. The cellular processes group contained transport and catabolism metabolic pathways, while the organismal systems group included environmental adaptation metabolic pathways (Fig 4).

### Expression analysis of genes encoding key enzymes for malic acid metabolism

Mature *C*. *humilis* and *C*. *glandulosa* fruits contained more malic acid than citric acid (Table 2), implying that malic acid is the main organic acid responsible for the differences in taste. The expression levels of genes encoding key enzymes involved in organic acid metabolism during different fruit-developmental stages were analyzed based on the transcriptome sequencing results and homologous sequences in the NCBI database.

**Fig 5:**
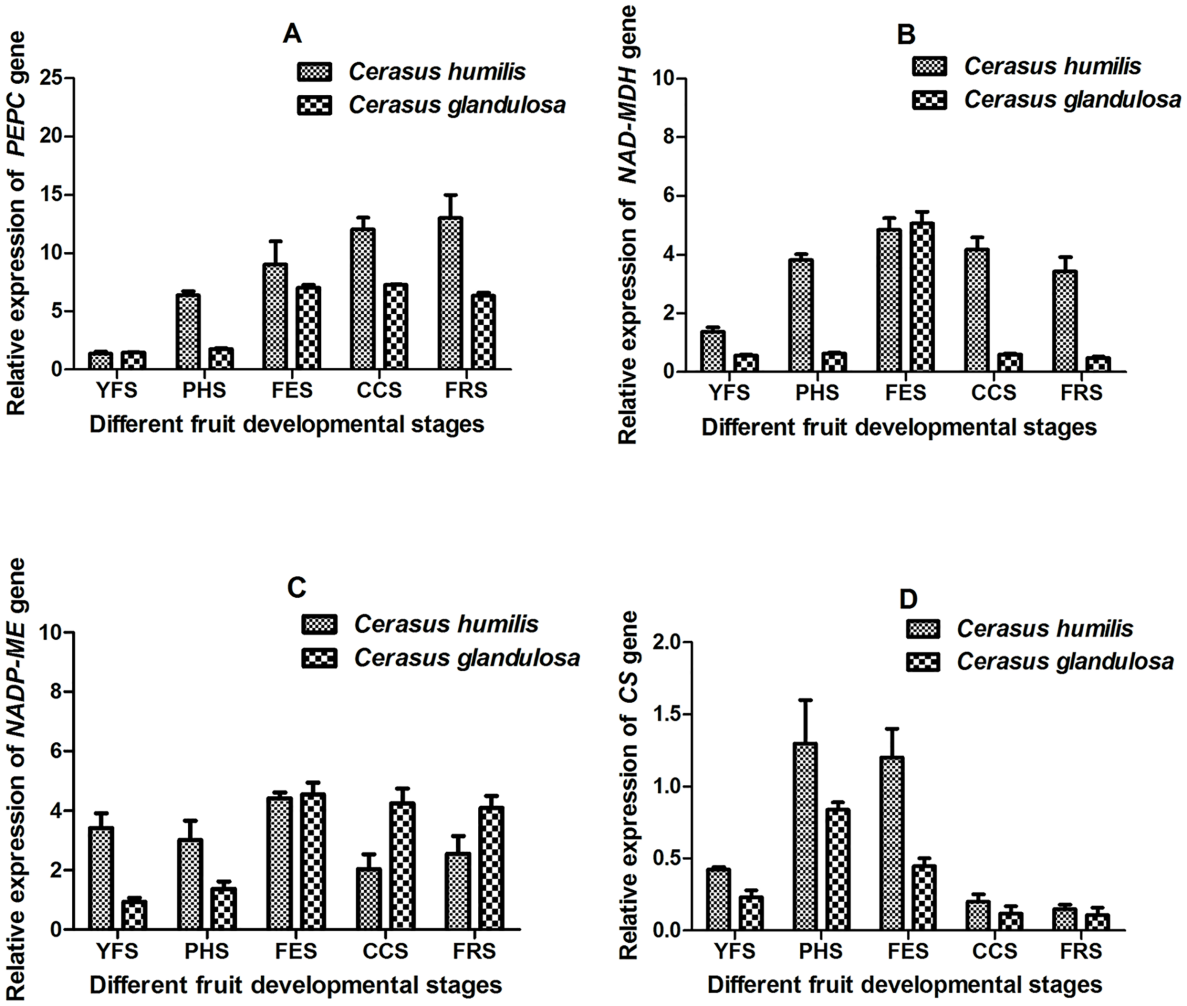
Changes in the *PEPC* (A), *NAD-MDH* (B), *NADP-ME* (C) and *CS* (D) gene expression levels in *C*. *humilis* and *C*. *glandulosa* during fruit development. YFS: young fruit stage; PHS: pit-hardening stage; FES: fruit-enlargement stage; CCS: color changing stage; FRS: fruit-ripening stage.

In *C*. *humilis* fruits, the *PEPC* gene expression level increased slightly from the young fruit stage (YFS) to the pit-hardening stage (PHS), followed by a rapid increase from the PHS to the fruit-enlargement stage (FES) (Fig 5A). The highest and lowest *PEPC* gene expression levels were observed at the FES and the color changing stage (CCS), respectively. The *PEPC* gene had a similar expression trend in *C*. *glandulosa* fruits. The *PEPC* gene expression level at the FES was much lower in *C*. *glandulosa* fruits than in *C*. *humilis* fruits. In contrast, at all other fruit-development stages, the *PEPC* gene expression level was slightly higher in *C*. *glandulosa* fruits than in *C*. *humilis* fruits. These results implied that the *PEPC* gene affected the accumulation of organic acids in *C*. *humilis* fruits primarily at the FES (Fig 5A).

In *C*. *humilis* fruits, the expression of the *NAD-MDH* gene decreased slightly from the YFS to the PHS, and then increased rapidly to reach the maximum value at the FES, which was followed by a rapid decrease at the CCS and a drastic increase at the FRS (Fig 5B). The *NAD-MDH* gene was highly expressed at the FES and at the FRS, with expression levels that were 5.58 and 7.42 times that of fruits at the YFS, respectively. The lowest expression level observed at the PHS was 0.81 times that of fruits at the YFS. Meanwhile, the highest *NAD-MDH* gene expression in *C*. *glandulosa* fruits was found at the FRS, which was 9.05 times that of fruits at the YFS, and the lowest *NAD-MDH* gene expression was observed at the PHS (Fig 5B). The *NAD-MDH* gene expression level was higher in *C*. *humilis* fruits than in *C*. *glandulosa* fruits throughout the fruit-developmental stage, but there was relatively little difference prior to the FES.

In *C*. *humilis* fruits, the *NADP-ME* gene expression level was the highest at the FES (2.17 times that of fruits at the YFS) and the lowest at the PHS (0.83 times that of fruits at the YFS). Meanwhile, in *C*. *glandulosa* fruits, the *NADP-ME* gene expression level was the highest at the FES (4.55 times that of fruits at the YFS) and the lowest at the YFS. Overall, the *NADP-ME* gene was more highly expressed in *C*. *glandulosa* fruits than in *C*. *humilis* fruits, except at the CCS. The relatively high *NADP-ME* gene expression levels induced the degradation of malic acid, especially at the FES and at the FRS (Fig 5C). The *Citrate synthase* (*CS*) gene expression levels were similar in two species and the *CS* gene was more highly expressed in *C*. *humilis* fruits than in *C*. *glandulosa* fruits (Fig 5D).

### Correlation analysis and principal component analysis

**Fig 6:**
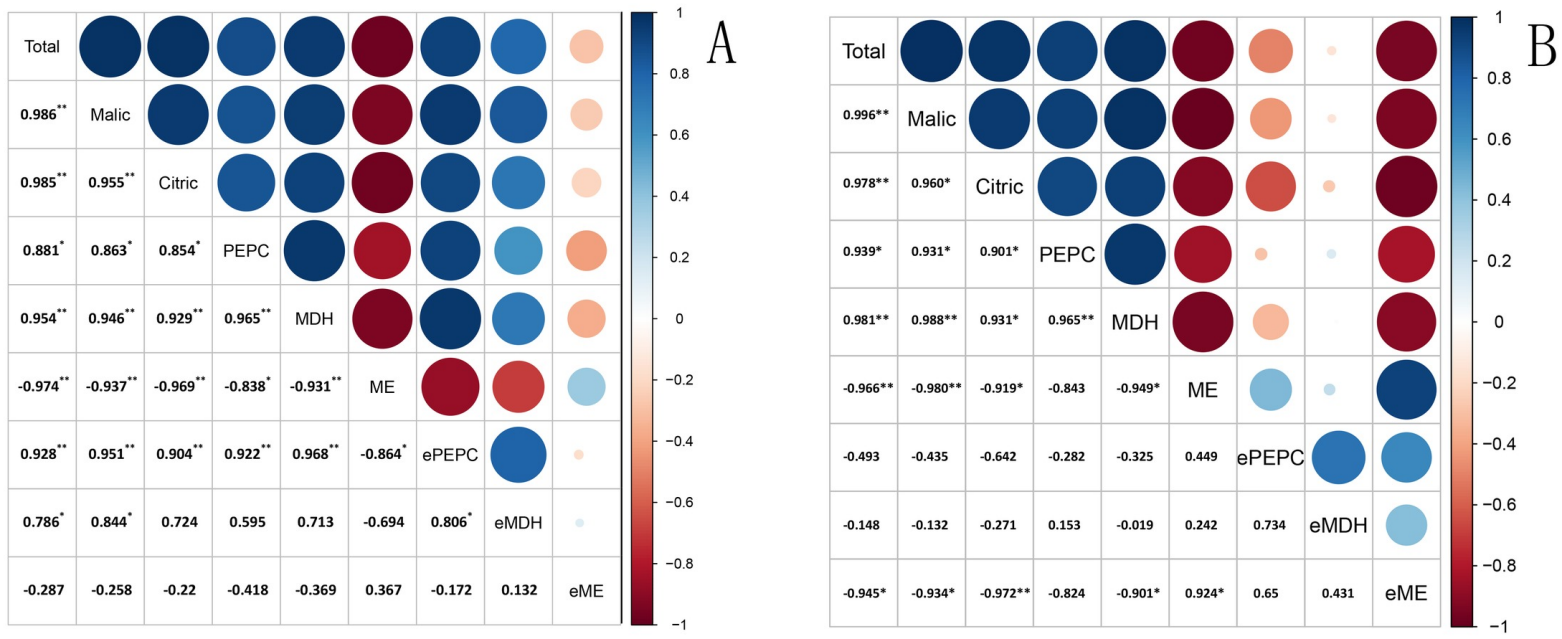
The correlation analysis of organic acid content, enzyme activity and the gene expression in developing fruits of *C*. *humilis* (A) and *C*. *glandulosa* (B). ** represents p< 0.01; * represents p< 0.05.

The correlation between gene expression, enzymatic activity and organic acid content in developing fruits of *C*. *humilis* and *C*. *glandulosa* was analyzed. Strong correlations are apparent among total acid, malic acid, and citric acid content in both *C*. *humilis* and *C*. *glandulosa* fruits (Fig 6). The *p*-value was the probability and was used to compare the confidence coefficient among the data groups in the the paired Tukey’s-test [37]. Significant correlation between data groups is indicated if *p*-value is less than 0.05 (p < 0.05). In this study, the levels of total acid, malic acid, and citric acid contents are all less than 0.05, indicating significant correlations. The correlation of organic acid contents with enzyme activities and the gene expression is also shown in Fig 6. PEPC and NAD-MDH are significantly positively correlated with the contents of organic acid while NADP-ME is significantly negatively correlated with the organic acid contents, PEPC and NAD-MDH in both *C*. *humilis* and *C*. *glandulosa* (Fig 6A and 6B).

In *C*. *humilis* fruits, the expression of *PEPC* gene is significantly positively correlated with organic acid contents and enzyme activity of NAD-MDH while it is significantly negatively correlated with the expression of the *NADP-ME* gene (Fig 6A). The expression of *NAD-MDH* gene is significantly positively correlated with total acid, malic acid and *PEPC* expression, but its correlations with citric acid content, PEPC activity, NAD-MDH activity and NADP-ME activity are not significant. The correlations between the expression of *NADP-ME* gene and other data are not significant which indicate that *NADP-ME* gene doesn’t have a decisive effect on the organic acid accumulation in the fruits of *C*. *humilis*. However, *NADP-ME* gene plays a very important role in the organic acid metabolism of *C*. *glandulosa* fruits. For example, the *NADP-ME* gene expression is significantly negtively correlated with the organic acid contents and enzyme activity of NAD-MDH.

The PCA results identified four components that explained 100% of the total variation in organic acid metabolism of *C*. *humilis* and *C*. *glandulosa* (Table 3). The first principal component (PC1) contributed 99.65% and 91.85% of the total variation in organic acid metabolism of *C*. *humilis* and *C*. *glandulosa* respectively, suggesting that the variation in PC1 could explain the differences in organic acid metabolism of these two species. In *C*. *humilis*, *NADP-ME* gene has the largest eigenvalue meaning that the *NADP-ME* gene plays the most important part in the organic acid metabolism of the fruits. In *C*. *glandulosa*, *NAD-MDH* gene and *NADP-ME* gene have the largest and the second largest eigenvalues respectively, suggesting that in addition to *NAD-MDH* gene, *NADP-ME* gene also has a substantial effect on the organic acid metabolism.

**Table 3:** Eigenvectors and eigenvalues of the first four principal components grouping the factors attributing to the organic acid metabolism in *C*. *humilis* and *C*. *glandulosa* fruits.

Principal components	*C*. *humilis*				*C*. *glandulosa*			
	PC1	PC2	PC3	PC4	PC1	PC2	PC3	PC4
Total acid	-1.295	-0.048	-0.051	0.029	-1.036	0.147	0.018	0.060
malic	-1.343	-0.045	-0.068	0.019	-1.122	0.126	-0.002	0.050
citric	-1.543	-0.096	-0.064	0.015	-1.479	0.106	0.013	0.044
NAD-MDH	0.471	0.582	0.129	0.033	**4.399**	0.916	-0.046	0.010
NADP-ME	-0.448	-0.485	0.150	-0.035	**2.375**	-1.547	-0.011	0.009
PEPC	-0.756	-0.036	0.073	0.006	1.085	0.184	0.123	-0.052
*NAD-MDH*	**6.774**	-0.125	-0.045	0.006	-1.476	0.070	0.009	-0.064
*NADP-ME*	-1.514	-0.123	-0.060	0.008	-1.386	-0.017	-0.015	0.012
*PEPC*	-0.345	0.376	-0.064	-0.081	-1.359	0.016	-0.089	-0.069
Eigenvalue	6.976	0.020	0.003	0.001	4.579	0.415	0.003	0.002
Proportion %	99.65	0.29	0.04	0.02	91.85	0.08	0.001	0.001
Cumulative %	0.9965	0.9994	0.9998	1	0.9185	0.9988	0.9995	1

## Discussion

To the best of our knowledge, we systematically report for the first time, the comparative study on the organic acid accumulation in fruits of *C*. *humilis* and *C*. *glandulosa*. In this study, the malic acid and citric acid content in *C*. *humilis* fruits accounted for 85.34% and 10.98% of the total acid content, respectively. In contrast, the malic acid and citric acid content in *C*. *glandulosa* fruits accounted for 88.90% and 4.74% of the total acid content, respectively. These results suggest that malic acid and citric acid are the main organic acids in both fruits, and likely affect fruit taste. The organic acid contents decreased in mature fruits, which is consistent with the results of earlier studies on nectarine, pear, and apple [6,7,18]. Additionally, the organic acid content decreased continuously throughout the fruit-developmental stages of *C*. *glandulosa*. However, in the developing fruits of *C*. *humilis*, the organic acid content increased gradually until one week before maturation. Although it underwent a sharp decrease in the fruit-ripening stage, the organic acid content in the fruits of *C*. *humilis* was still high, making the fruits very sour.

In our study, 6,594 differentially expressed genes were detected based on transcriptome sequencing results, including 12 genes involved in malic acid metabolism, 25 genes involved in the citric acid cycle, 194 genes related to NAD/NADP metabolism, and one PEPC gene. These genes may be involved in the biosynthesis or catabolism of organic acids in *C*. *humilis* and *C*. *glandulosa* fruits, with potential consequences for fruit quality. There are at present relatively few reports describing the transcriptional regulation and cloning of genes regulating organic acid contents in *C*. *humilis* and *C*. *glandulosa* fruits. Therefore, the differences in gene expression levels described herein may be useful for future studies.

Through correlation analysis, we discovered strong correlations between organic acid content, enzyme activity and gene expression in both *C*. *humilis* and *C*. *glandulosa* fruits. First of all, PEPC, NAD-MDH and NADP-ME are significantly correlated with the organic acid contents in both *C*. *humilis* and *C*. *glandulosa*. In addition, *NADP-ME* gene doesn’t have a decisive effect on the organic acid accumulation in *C*. *humilis* while it plays a very important role in the organic acid metabolism of *C*. *glandulosa*. The PCA analysis was carried out and the results verified the important role of *NADP-ME* gene in the organic acid metabolism in *C*. *glandulosa*.

Our results suggest that NAD-MDH, NADP-ME, and PEPC are all involved in regulating organic acid biosynthesis in *C*. *humilis* and *C*. *glandulosa* fruits. However, the gene expression levels were not directly related to changes in malic acid content.

## Supporting information

S1 Table List of all unigenes obtained through transcriptome sequencing of *Cerasus humilis* and *Cerasus glandulosa*. (XLS)

S2 Table List of differentially expressed unigenes between *Cerasus humilis* and *Cerasus glandulosa* which were related to organic acid metabolism. (XLS)
